# ‘It Should Be All Hands on Deck’: A Qualitative Exploration of Shared Experiences and Needs of Older Adults, Family Carers, and Health Professionals in Disability Management in China

**DOI:** 10.1155/jonm/6577817

**Published:** 2026-10-11

**Authors:** Yitong Mao, Zhiting Guo, Yajun Ma, Yuping Zhang, Jingfen Jin

**Affiliations:** ^1^ Nursing Department The Second Affiliated Hospital of Zhejiang University School of Medicine (SAHZU) Hangzhou Zhejiang 310009 China; ^2^ Key Laboratory of the Diagnosis and Treatment of Severe Trauma and Burn of Zhejiang Province Hangzhou China; ^3^ Zhejiang Provincial Clinical Research Centre for Emergency and Critical Disease Hangzhou China

**Keywords:** disability, ecological systems theory, family carers, health professionals, older adults, qualitative

## Abstract

**Background:**

Population ageing has increased the demand for disability care services in China. Disability in older adults is associated with adverse health outcomes and therefore requires a collaborative approach involving hospitals, communities and families. However, the collaborative experiences and needs of key stakeholders—older adults, family carers and health professionals—remain underexplored from a multifaceted approach, hindering the development of integrated care systems.

**Objective:**

To explore the experiences, perceived challenges and collaborative needs of older adults, family carers and health professionals about disability management in China.

**Methods:**

We conducted a descriptive qualitative study using semistructured, in‐depth interviews. A total of 44 participants (17 older adults, 11 family carers and 16 health professionals) were recruited from hospitals and communities. Data were analysed using an inductive thematic framework approach guided by Bronfenbrenner’s ecological systems theory.

**Results:**

Key themes were identified across four ecological levels: (1) microsystem: insufficient awareness and excessive burden; (2) mesosystem: cross‐agency care coordination and interprofessional integration; (3) exosystem: policies and allocation of social resources; and (4) macrosystem: the dual influence of filial piety culture.

**Conclusion:**

Disability management for older adults in China is a complex, multilevel challenge anchored not only in resource deficits but also in a shared disability literacy deficit and culturally reinforced care dependencies. Filial piety emerged as a dual force that sustains family care while masking care needs and straining carers. Realising integrated care requires a shift from fragmented, reactive services to an interdependent, function‐oriented continuum, with specialist nurses acting as pivotal coordinators across hospital, community and home. Future interventions and policy should prioritise multilevel frameworks that embed routine functional assessment and targeted caregiver capacity‐building to support sustainable healthy ageing.

**Key Points**
• This study explores the shared experiences, challenges and needs of older adults, family carers and health professionals in disability management in China through a multistakeholder, ecological‐systems lens.
• The study identifies systemic barriers at four ecological levels: microsystem (insufficient awareness and excessive burden), mesosystem (cross‐agency care coordination and interprofessional integration), exosystem (policies and allocation of social resources), and macrosystem (the dual influence of filial piety culture).
• Translating the findings into practice requires a shift from fragmented, reactive care to an interdependent, function‐oriented continuum of care, with specialist nurses empowered as pivotal coordinators across hospital, community and home.
• Future interventions and policy should prioritise integrated, multilevel frameworks that embed routine functional assessment and targeted caregiver capacity‐building to support sustainable healthy ageing.

## 1. Introduction

Population ageing is a significant demographic phenomenon resulting from declining fertility and increasing longevity [1]. The World Health Organization (WHO) predicts that by 2050, two‐thirds of the world’s population over 60 years will live in low‐ and middle‐income countries [2]. According to the latest national statistics, individuals aged 60 years and above have reached 296.97 million by 2023 in China, accounting for 21.1% of the total population [3]. This demographic reality is inextricably linked to an increasing frequency of chronic conditions, functional decline and disability among older adults [4]. Disability is typically defined as limitations in basic activities of daily living (BADLs) and/or instrumental activities of daily living (IADLs) due to physiological, psychological or social factors [5, 6]. Most individuals experience a decline in health or a gradual loss of functional ability as they age, making it difficult to perform valuable daily activities such as bathing or housekeeping [7]. The prevalence of disability among older adults in China is approximately 26.4%, higher than in the United States (15.6%) and the United Kingdom (18.3%) [8]. A higher disability rate is typically associated with negative outcomes such as depression, decreased quality of life, increased hospitalisation and mortality risks [9–11], resulting in large long‐term care needs.

However, caring for the disabled is an incredibly complicated and strenuous task [12]. For hospitals, the disease‐centred healthcare model prioritises treating acute diseases over providing preventive services [13, 14]. Due to Chinese filial piety culture, older adults typically prefer to age in place [15], so they usually return to communities and families after treatment. In China, as in many other low‐ and middle‐income countries, children and spouses bear primary responsibility for older adults’ financial and physical care [16]. Therefore, coordinated efforts across multiple settings are necessary to support older adults with or at risk of disability. This calls for a shift from fragmented care to integrated disability management. Although this term originated in occupational health, where it denotes a systematic and collaborative effort to minimise disability and its consequences [17], it is equally applicable to older adults, whose functional decline unfolds gradually and across settings. We build on two established formulations: the disablement process, which regards functional decline as a modifiable intermediate stage rather than an inevitable endpoint of disease [18]; and the WHO’s function‐centred reframing of healthy ageing, which orients services toward intrinsic capacity (IC) and functional ability rather than discrete diagnoses [6, 13]. Accordingly, we adopt the following working definition: Disability management is a systematic, collaborative process that aims to prevent or mitigate declines in functional ability among older adults. Unlike typical geriatric care, which is frequently disease‐centred, it is proactive, coordinated and function‐orientated, spanning prevention, early intervention and long‐term support across hospital, community and home settings.

Several relevant studies have examined the care experiences and needs of different stakeholders separately. For instance, studies have explored the experiences of formal carers [19], dyadic interaction experiences between people with disabilities and their carers [20] and the significant stress and role ambiguities faced by spousal carers in long‐term care settings [21]. The subjective experiences of older persons with disabilities have also been the focus of some research [22, 23]. The contact patterns and influencing factors between community nurses and family carers have been examined in other studies [24]. Although these studies have enhanced our understanding of interactions from single parties or between two parties, the collaborative dynamics among older adults, carers and professionals remain unclear. To methodically comprehend these intricate relationships, this study adopts Bronfenbrenner’s ecological systems theory as an analytical framework [25]. It emphasises multiple levels—from interpersonal interactions to cultural values—making it better suited to the Chinese context where disability management is shaped by family dynamics, resource allocation and filial piety. We will systematically explore the experiences, perceptions and collaborative needs regarding disability management among older adults, family carers and hospital and community professionals using a multistakeholder qualitative study approach.

## 2. Method

This study employed a qualitative descriptive design to capture and describe the experiences and perceptions of different populations (older adults, family carers and health professionals) about disability management, adhering to the general principles of naturalistic inquiry [26, 27]. The study process and results are reported based on the Consolidated Criteria for Reporting Qualitative Studies (COREQ) [28] checklist (Appendix S1).

### 2.1. Study Setting, Recruitment and Inclusion/Exclusion Criteria

To gain a better understanding of different regions, the participants were recruited from three tertiary general hospitals and four communities (health service centres providing primary healthcare and public health services) in Hangzhou City and Huzhou City, Zhejiang Province, China. Purposive sampling and maximum variation sampling methods were used to recruit older adults aged over 65 years and health professionals [29]. The researchers carefully selected participants with a variety of characteristics (e.g., age, gender, level of disability for older adults and work experience and job title for professionals) to maximise the potential diversity of perspectives. Snowball sampling was used to find primary carers for older persons who gave their consent. Table 1 displays the participants’ inclusion and exclusion criteria.

**TABLE 1:** The inclusion and exclusion criteria.

The criteria for the inclusion and exclusion
For older adults
Inclusion criteria
Age greater than or equal to 65 years
Exclusion criteria
Having severe cognitive impairment or hearing limitations
For health professionals
Inclusion criteria
1. Age greater than or equal to 18 years
2. A health professional (physician or nurse) actively working in a geriatric ward or community health centre for at least 3 years
3. Self‐reported that they routinely care for patients with disabilities
Exclusion criteria
Unable or reluctant to cooperate with the interview
For family carers
Inclusion criteria
1. Age greater than or equal to 18 years
2. Family members of older adults and primarily responsible for the daily care of the older person
Exclusion criteria
Unable or reluctant to cooperate with the interview

Disability was assessed using BADLs. The BADL measures the respondent’s ability to perform basic daily tasks, including dressing, bathing, eating, getting out of bed, using the toilet and controlling urination and defecation [30]. This study focused on BADL because loss of basic self‐care ability more directly leads to caregiving dependence and health service utilisation, which are the central concerns of disability management. Having no difficulties in BADL is regarded as a sign of independent older adults. Mild disability is defined as one to two difficulties, moderate disability as three to four difficulties and severe disability as five to six difficulties [30]. The disability level was determined by the research team based on self‐reported information from the older adults or their family members.

The demographics of interviewed participants are shown in Table 2. Overall, 44 participants (17 older adults, 11 family carers and 16 health professionals) participated in interviews. Three eligible individuals (1 older adult and 2 family carers) declined participation due to time constraints. There is a more balanced distribution of different levels of disability among older adults. The majority of health professionals interviewed are females and nurses. Family carers include both spouse carers and child carers. The majority of carers (72.7%) are female, reflecting the gendered realities of caregiving in China, where women primarily assume familial care responsibilities. This composition allows for a more in‐depth exploration of women’s caring experiences but may also mean that the viewpoints of male carers are underrepresented. These features were addressed throughout data analysis, with a focus on how systemic and cultural issues influenced the reported experiences.

**TABLE 2:** Interviewed participants’ demographics.

	Older adults^∗^ *n* = 17	Family carers^∗^ *n* = 11	Health professionals^∗^ *n* = 16
Sex, *n* (%)			
Male	10 (58.8)	3 (27.3)	2 (12.5)
Female	7 (41.2)	8 (72.7)	14 (87.5)
Age in years, mean *(SD)*	80.35 (11.0)	68.1 (13.0)	36.4 (7.3)
Level of disability			
No disability	3 (17.7)		
Mild disability	5 (29.4)		
Moderate disability	5 (29.4)		
Severe disability	4 (23.5)		
Type of carers			
Spouse carer		6 (54.5)	
Child carer		5 (45.5)	
Care duration years, mean (SD)		6.41 (5.02)	
Profession			
Physician			6 (37.5)
Nurse			10 (62.5)
Workplace			
Hospital			11 (68.8)
Community			5 (31.2)
Profession title			
Junior			4 (25)
Middle			7 (43.8)
Senior			5 (31.2)

^∗^All participants are Chinese.

### 2.2. Data Collection

Two researchers (Y.M._1_ and Z.G.) collected the data between December 2023 and January 2024. Kallio et al.’s methodologies were used to develop a topic‐based interview guide with semistructured open‐ended questions [31]. A pilot interview was conducted with 3 health professionals, 1 older adult and 1 carer to test the interview guide. The interview guide was modified by the research group (Y.M._2_, Y.X. and J.J.) with expertise in geriatric care and qualitative research, as shown in Appendix S2. Before each interview, the researchers familiarised themselves with the environment and allowed ample time for a preinterview conversation to explain the study, answer questions and establish a respectful, collaborative tone. All interviews were conducted one‐on‐one, face‐to‐face in Mandarin, the official language of China. Interviews were conducted in a quiet, private meeting room with only participants and researchers present. Interviews lasted 35 min on average, and field notes were taken. Data collection continued until saturation was judged to have been reached. Saturation was defined following Saunders et al. [32], as the point at which two consecutive interviews within each participant group yielded no new codes and no substantively new dimensions of existing codes. The absence of new codes in successive interviews was determined by research group consensus. There were no repeated interviews.

### 2.3. Data Analysis

All interviews were recorded and transcribed verbatim into Chinese. Transcripts were translated into English by the first author, a bilingual researcher. Subsequently, another researcher back‐translated the English transcriptions into Chinese to ensure accuracy. The research group discussed and reached consensus on some words and expressions that may have differences in cultural context. During transcription and translation, potentially identifying details (e.g., specific diagnoses and care settings) were removed or generalised where they were not essential to the meaning of the quotation. All data were analysed using an inductive thematic framework. Transcripts were coded and categorised into themes according to Braun and Clarke’s six‐step framework [33]. To capture all important information, codes were created inductively with an open coding method. Two researchers (Y.M._1_ and Z.G.) independently coded and extracted the themes using NVivo 12 Plus software (QSR International). Disagreements between the two independent coders were resolved through consensus discussion. When consensus could not be reached, a third researcher was consulted. The research group determined the final themes by a consensus‐building process. During the interpretative phase, Bronfenbrenner’s ecological systems theory provided a framework for mapping emerging themes to micro‐, meso‐, exo‐ and macro‐level influences. Participants did not provide feedback on the final themes. Participants in the pilot study were not included in the final sample and analysis.

### 2.4. Rigour and Reflexivity

The COREQ checklist [28] was used for quality assurance. To ensure dependability and confirmability, audio recordings and independent data analysis were employed [34]. The interviews were conducted by two unbiased researchers who had no prior relationships or conflicts of interest with the participants. The interview guide was developed methodically and refined by the entire research team, which enhanced the study’s credibility. Maximum variation sampling was used to improve participant representation and transferability. In order to ensure that the transcripts appropriately represented their thoughts and feelings, participants also went over parts of the transcripts for confirmation, clarification or additional information.

In terms of the personalities of the researchers, the principal interviewers (Y.M._1_ and Z.G.) were both nursing PhD students with backgrounds in gerontology and qualitative research. We acknowledge that gender, age and professional identity may influence the research process. For example, these factors might make it easier to empathise with female participants. To mitigate such biases and enhance the credibility of the analysis, the entire research team participated in theme review and refinement after the extraction of themes through dual independent coding. This made sure that the themes stayed rooted in the initial data.

### 2.5. Ethical Considerations

Ethical approval for this study was obtained from the Human Research Ethics Committee of the Second Affiliated Hospital of Zhejiang University School of Medicine (No. 20231268). Participants provided written informed consent, with the option to withdraw at any time. All data were anonymised and stored in password‐protected files.

## 3. Results

The experiences of older adults, family carers and professionals during disability management were divided into four themes and six subthemes based on social ecosystem theory (Table 3). In the ecological framework of disability management, challenges at the microsystem level include insufficient awareness and excessive care burden. Themes at the mesosystem level stem from collaboration between hospitals and communities. Resource allocation within the exosystem is particularly crucial. Additionally, disability management is influenced by traditional filial piety culture, which functions as a macrosystem. Figure 1 illustrates the ecological framework of disability management and the themes identified at each level.

**TABLE 3:** Thematic table of themes and subthemes.

Ecological systems	Themes	Subthemes	Primary stakeholder(s)^∗^
Microsystem	Insufficient awareness and excessive burden	Knowledge and attitude gaps regarding disability	①②
		Excessive care burden	②

Mesosystem	Cross‐agency care coordination and interprofessional integration	Care coordination between home and hospital	①③
		Interdisciplinary professional team‐building	①③

Exosystem	Policies and allocation of social resources	Community resource allocation	①③
		Involvement of social resources	②③

Macrosystem	The dual influence of filial piety culture	/	①②

^∗^① Older adults; ② family carers; ③ health professionals.

**FIGURE 1:**
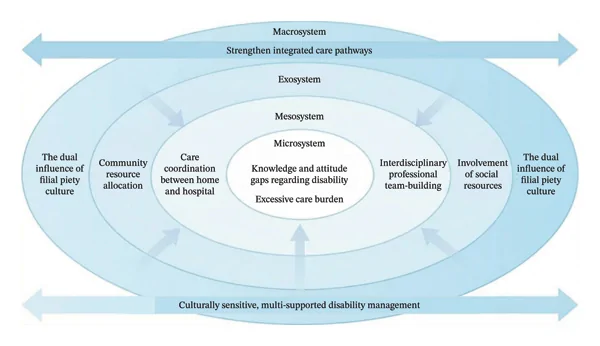
Overview of themes and subthemes across ecological system levels.

Both older adults and family carers lack sufficient understanding of disability. Older adults often downplay the concept of disability and see functional decline mainly as a result of ageing or sickness. Family carers highlight the excessive burden of care and the emotional and financial pressures of daily caregiving and the practical consequences of filial duty expectations. Health professionals concentrate more on systemic limitations, such as gaps in policy implementation, insufficient community resources and fragmented cross‐sectoral collaboration. Appendix S3 contains a few more typical quotes from the participants.

### 3.1. Microsystem: Insufficient Awareness and Excessive Care Burden

#### 3.1.1. Knowledge and Attitude Gaps Regarding Disability

It is rare for older adults to view disability as a manageable continuum. Most older adults concentrate on urgent and tangible health issues rather than disability prevention. Notably, the terms ‘disease’ and ‘disability’ were often confused by older adults. One stated that: *‘I’m currently suffering from complications related to a chronic condition, and my injury prevents me from walking.’ (C16, older adult, moderate disability).* Even when older adults were aware of functional decline, they did not regard it as a disability, such as, *‘I’m not disabled; I’m simply getting older, which makes me walk more slowly and have trouble climbing stairs.’ (C17, older adult, mild disability).* Furthermore, they frequently adopt a subservient or negative attitude and concentrate primarily on the current physical sensation: *‘There’s not much that bothers me… I haven’t thought about the future either. I have no idea what′s going to happen in the future, so it’s useless to think about it. I′m simply living each day as it comes.’ (C1, older adult, no disability).*

Family carers’ knowledge of disability is frequently limited to basic daily support, such as the requirement for help eating and walking. One spouse carer remarked, *‘Every day I just wash his face, feed him, and turn him over – I don’t know what else to do’ (D9, spouse of an older adult with moderate disability)*. Consequently, the daily care provided by carers often lacks preventive training or rehabilitation exercises. A daughter caring for her bedridden mother said, *‘Because of her wounds, we can’t move her around freely for fear of causing her pain, so we just try to keep her lying comfortably.’ (D14, child of an older adult with severe disability)*. Gaps in carers’ knowledge and skills may further limit their ability to take proactive measures to prevent disability. One mentioned, *‘(I’ll) squeeze his legs. It’s probably not even a massage. I have no specific knowledge or anything. I only want to make him feel better.’ (D9).*

#### 3.1.2. Excessive Care Burden

Carers often experience a range of considerable challenges: health‐related pressures, financial pressures and psychological pressures, particularly those caring for the disabled. Many carers, especially those who are older, find it difficult to strike a balance between care responsibilities and their own health needs. One spouse carer expressed this strain frankly: *‘I don’t have the strength anymore — it’s too much. He’s old, and so am I; I also need to take care of myself*.*’ (D6, spouse of an older adult with severe disability)*. Financial pressures further exacerbate these challenges, especially when out‐of‐pocket medical expenses are high. One carer noted that *‘he needed to spend a substantial share of the household’s monthly income on medication, all out of pocket. Some drugs aren’t well reimbursed and are very expensive, but he has to take them every day*.’ *(D9).*

Adaptability challenges and role conflict also exacerbate the caregiving load. Inexperienced or unprepared carers are sometimes put into complex care tasks with little assistance, particularly following a sudden decline in the older adult’s health. As one child carer described, *‘I had nothing to worry about while she was well. But now, I have no choice—I have to learn (how to care for her).’ (D4, child of an older adult with moderate disability)*. In particular, female carers often balance intergenerational duties, taking care of younger children and elderly parents while also managing their own well‐being. Overall, these carers, especially those caring for the disabled, encounter tremendous care burdens and challenges.

### 3.2. Mesosystem: Cross‐Agency Care Coordination and Interprofessional Integration

#### 3.2.1. Care Coordination Between Home and Hospital

Several respondents emphasised the importance of care coordination between home and hospital. Older adults highlighted the lack of discharge guidance, while health professionals focused on structural hurdles like confusing referral systems and insufficient capacity for community follow‐up. Older adults are typically admitted to the hospital for acute illnesses and then discharged. After discharge, there appears to be a ‘window period’ during which there are no nursing interventions until readmission. As one interviewee mentioned, *‘After I was discharged last time, I still didn’t know how to exercise (to build strength in my lower body), and I ended up falling over again… The doctor told me to do more exercise, but no one taught me exactly how to do it.’ (C5, older adult, mild disability).* It is clear that cooperation between communities and hospitals still needs to be strengthened. Hospital professionals have recognised the importance of continuity of care for older adults. However, the poor connection between communities and hospitals may lead to less effective disability management: *‘I believe one issue is that it has not yet been fully extended to the community. If the community could provide ongoing support (monitoring and follow-up) after he (the older adult) arrives, efforts to prevent and manage disability would be more effective.’ (A10, a hospital nurse).* Communities also paid little attention to collaborating with hospitals: *‘We’re still mainly managing hypertension and diabetes. Well, knowing the disability status of older adults is also unintentional, because there’s no module specifically set up to manage it.’ (B1, a community physician)*.

#### 3.2.2. Interdisciplinary Professional Team‐Building

The need for interdisciplinary collaboration was primarily raised by health professionals in both hospital and community settings. In China, geriatric units and speciality divisions like nutrition and rehabilitation frequently operate independently, leading to fragmented care. Staff members may only participate in regular rounds or ad hoc consultations even when they are assigned to geriatric departments, which limits their capacity to regularly track functional changes. As one hospital nurse stated, *‘It would be better to have therapists under our geriatrics department. In certain cases, such as our morning handover, we are well aware of the patient’s circumstances, but the therapist may not be as clear. They are simply carrying out their duties, and we are carrying out ours.’ (A9, a hospital nurse).* Similarly, a community nurse highlighted coordination challenges: *‘The current composition of the community staff primarily consists of physicians and nurses; the inclusion of specialist physiotherapists and counsellors would be beneficial.’ (B4, a community chief nurse).*

The geriatric team’s expertise was also emphasised. The professionals pointed out that in order to give disabled people better medical care, several subspeciality‐focused doctors and nurse specialists should be involved. *‘An interdisciplinary team is needed. A subspeciality orientation is also necessary for the medical staff′s composition. Some individuals have a psychiatric focus… Eventually, it becomes an interdisciplinary team focused on the disabled.’ (A10).* Moreover, the collaboration of the interdisciplinary geriatric team was crucial, which may affect the effectiveness of medical services provided to older adults. *‘The team members’ coordination is not good now. For example, physicians may not think of working with other treatments if a single patient exhibits any symptoms. It’s poorly integrated.’ (A1, a community nurse).* Older people and their carers also emphasised the need for specialised care within the community setting. For example, one older adult described their experience with a community clinic: ‘*We tried visiting community clinics, but when they saw my wounds, they remarked, ‘We’ve never seen such a severe case.’. They lacked the necessary qualifications. Ultimately, we still required hospital professional nurses to manage it.’ (C15, older adult, severe disability)*.

### 3.3. Exosystem: Policies and Allocation of Social Resources

#### 3.3.1. Community Resource Allocation

Community health professionals mentioned the issue of community resource allocation, including human resources and environmental facilities. One nurse said, ‘*We have a heavy workload: a large number of people come every day for medication. We also need to measure blood sugar and blood pressure and make home visits.’ (B3, a community nurse).* Some demands may be unfulfilled due to inadequate staffing and an excessive workload, which could result in poor disability management. Currently, the education level of community professionals is primarily specialised and undergraduate, with a notable lack of postgraduate staff. This may make it difficult to gain the trust of older adults when dealing with complex conditions: *‘Some complex cases may require the involvement of a specialist, such as a physician with a postgraduate degree.’ (B5*, *a community chief nurse).* Additionally, community professionals indicated that the absence of facilities and equipment made it difficult to undertake disability interventions. One suggested that ‘*I would suggest that it would be more beneficial to have a place with necessary facilities where they can do exercise and rehabilitation.’ (B4).* The absence of proper supplies in the community was also brought up by an older adult: *‘The catheter needed to be replaced at the hospital every few weeks, but they couldn’t do it themselves and didn’t have the supplies.’ (C7, older adult, severe disability).*

#### 3.3.2. Involvement of Social Resources

Apart from hospitals, communities and families, social resource involvement was also considered a crucial element by health professionals and older adults. One aspect was the formulation, promotion and implementation of disability‐related policies at the national level. Although there were some policies in place at the time, they were not adequately implemented. One said that in order to have an effective overall plan, disability management needs to be directed nationwide. Health commissions and local governments then took successive action: *‘It may need the power of the national authority. For instance, the Health Commission spearheads health education initiatives to raise public awareness of geriatric syndromes, disabilities, and diseases in general. This allows for early identification and intervention.’ (A11, a hospital chief physician).*

Nursing institutions and social volunteers must also be taken into account. Older adults living in institutions depended on the daily care provided by nonrelative carers. Therefore, out‐of‐hospital disability management also included institutional care for older adults: ‘*It would be better if there were professional care homes… At the moment, we have no choice but to shoulder the burden ourselves (in caring for the older adult)*.*’ (D15, spouse of an older adult with severe disability).* Moreover, a community manager pointed out that volunteer participation was necessary for disability management: *‘It should be all hands on deck. The government and community volunteers need to link up to care for older adults. Volunteers also provide care for older adults, thus creating a harmonious atmosphere.’ (B5).*

### 3.4. Macrosystem: The Dual Influence of Filial Piety Culture

The influence of Chinese filial piety was discussed mainly by older adults and family carers, who described it as both a moral pillar and a source of stress. On the one hand, it forces spouses and children to take on long‐term care responsibilities, reinforcing the moral obligation of family caregiving. According to carers, this obligation is presented as an indisputable moral requirement: *‘A young couple’s companion in old age. I will be by his side for another year as long as I live. I will definitely take care of him if he becomes sick.’ (D18, spouse of an older adult with mild disability).* The care recipient’s expectations may also be shaped by this, leading them to favour family care over care from ‘strangers’: *‘I’d rather not have a carer assist me; I prefer someone I know to a stranger.’ (C14, older adult, severe disability).* On the other hand, filial piety can increase the physical and psychological strain on family carers, who typically place a higher priority on honouring their elders and taking care of their parents directly. *‘As her children, how could we possibly blame her for that? We just have to be patient and put up with it.’ (D10, child of an older adult with mild disability). ‘I’ve thought about hiring the carer, but it’s better to have someone I know and trust look after him. Besides, he wouldn’t feel at ease with a stranger around.’ (D11, child of an older adult with moderate disability).*

## 4. Discussion

From the viewpoint of several stakeholders based on ecological systems theory, this study investigated the experiences, perceptions and cooperative requirements of individuals participating in disability management across several settings. This study demonstrated the difficulties and needs of managing disabilities at the micro‐, meso‐, exo‐, and macrosystem levels within the theoretical framework of ecological systems theory. These systems are not isolated entities but are interdependent. Therefore, in order to effectively promote disability management, systemic impediments must be removed to facilitate positive interaction and resource integration across all levels.

### 4.1. Microsystem: Enhancing Disability Awareness and Supporting Family Carers

Microsystem refers to the immediate environments with which the individual interacts directly, which can be regarded as the cornerstone of disability management. Our research revealed that older adults and family carers had significant gaps in their knowledge and understanding of disability. Older adults often conceptualise disability as a static condition of incapacity rather than as a dynamic process that can be influenced by intervention. Instead of maintaining function, they frequently concentrate on urgent health issues. Similarly, family carers’ knowledge was often restricted to basic everyday assistance, with little knowledge of prevention or rehabilitative techniques. These misconceptions may stem from a lack of targeted health education and reflect a larger systemic disregard for functional ability and IC in standard care practices [35, 36]. Therefore, in order to improve their knowledge and abilities, it is necessary to synthesise existing evidence and establish a comprehensive knowledge system within a framework focused on functional disability and IC.

Moreover, family carers—mostly spouses and adult children—bear heavy physical, financial and psychological burdens. We found that more experiences were shared by those who cared for older adults with moderate to severe disabilities. It is possible that these older adults’ everyday activities were more limited, resulting in carers taking on more care responsibilities. These carers, who are described as unpaid, nonprofessional carers [37], frequently had to deal with role conflicts, a lack of caregiving expertise, and limited support systems. Even though they frequently struggled with their own age‐related health issues, spouses were usually the primary carers if they were healthy enough; some even put the needs of the older adult ahead of their own [38, 39]. In the meantime, child carers found it difficult to juggle the obligations of their jobs with providing care. It is more severe among female carers managing intergenerational responsibilities. In line with the gendered makeup of our sample and more general socioeconomic trends in China, women were primarily responsible for providing care, whether as adult daughters or wives. These findings are consistent with previous studies that highlighted the negative effects of carer burden on health and well‐being [40]. This underscores the urgent need for tailored support. Even though there are some successful strategies, such as family‐centred therapies [41], their applicability in domestic contexts requires further validation. Going forward, actions should improve the abilities of carers and provide care services to families. While models like PACE in the United States have demonstrated effectiveness in supporting both older adults and carers [42], their implementation in China may face logistical and financial challenges. The current health system allocates disproportionate resources to tertiary hospitals, and community‐based long‐term care financing remains limited. Therefore, any adaptation of PACE‐like models would require policy‐level investment, strengthened primary care capacity and sustainable reimbursement mechanisms. Furthermore, flexible short‐term care and rehabilitation can be provided by community‐based alternatives like day care, giving carers much‐needed relief.

### 4.2. Mesosystem: Strengthening Cross‐Sector Coordination and Interdisciplinary Collaboration

At the mesosystem level, our findings highlighted critical gaps in care coordination between hospitals and communities, as well as inadequate professional integration. The shift from the hospital to the home has been identified as a vulnerable period [43, 44], often characterised by inconsistent follow‐up and inadequate support [14]. Due to systemic staffing and resource problems [45], transitional care in China is still mostly restricted to telephone follow‐up and basic nurse‐led instruction [46]. Additionally, this study discovered that certain older adults need specialised nursing care, including catheter upkeep and wound dressing changes. These services currently rely highly on hospital‐based professional nurses. This exacerbates the existing care gap between hospitals and the community. Evidence‐based models such as the Care Transitions Program, which utilises trained nurses as transition coaches to empower older adults and families in self‐management [47], should be modified and scaled to address these discontinuities. Concurrently, digital health platforms could enhance information sharing between settings [48], supporting more seamless and standardised disability management. Efforts also should be made to explore ways of improving the community’s ability to handle specialised nursing needs through skill development and remote guidance.

Effective disability management requires a change from a multidisciplinary approach to true interdisciplinary teamwork [49], where professionals collaborate to carry out assessments, planning and interventions based on common functional goals. In these teams, clinical nurse specialists (CNSs), especially those with geriatric competence, can play a crucial role as coordinators, overcoming communication barriers and guaranteeing continuity of care in both hospital and community settings [50, 51]. Nevertheless, a large number of health professionals are not formally trained in collaborative practice [52]. There is a need for interprofessional integrated education, which should emphasise communication and collaborative skills based on principles [53]. By strengthening both structural coordination and relational integration, it is possible to create a more cohesive care ecosystem to support older adults’ functional health across various settings—from hospital to home.

### 4.3. Exosystem: Optimising Resource Allocation and Social Support Systems

According to the team effectiveness pyramid [54], a system’s ability to function effectively is based on its basic human and physical resources. However, due to a resource allocation system that has favoured higher‐level hospitals [40], China’s primary healthcare sector today faces a significant scarcity of skilled personnel, outmoded equipment and restricted service capacity [55]. This imbalance increases the gap between service provision and demand, erodes trust among older adults and undermines the quality of community‐based disability management. To address these structural inequities, resource reallocation must be strategically guided toward strengthening primary care. To attract and retain qualified professionals in grassroots settings, initiatives could include expanding training programmes to community health workers, improving infrastructure and offering incentives. Programmes like the US Geriatrics Workforce Enhancement Program provide a useful resource for expanding a multidisciplinary workforce that can adapt to the ageing population [56].

Policy‐level integration and promotion are equally crucial. China still lacks a national implementation plan for integrated care models, despite the fact that caring for the elderly has emerged as a worldwide policy priority [57]. Disability management involves collaboration across multiple sectors, including healthcare, rehabilitation and social services, which calls for strong interdepartmental policy coordination and top‐level planning [58]. Healthcare facilities and providers of senior care services have historically functioned independently [59], with significant information barriers. In the future, efforts should be made to promote the deep integration of medical care, elderly care and rehabilitation resources at the institutional, informational and service levels. Furthermore, enhancing community health and social services is crucial given the rising need for long‐term care among older adults [40]. Volunteer services can serve as a vital supplement, improving older adults’ well‐being and social support [59]. However, the current volunteer participation rate remains below actual needs [60]. Policy guidance is required to establish standardised, sustainable volunteer service mechanisms, enabling volunteers to become an indispensable social force within the disability care system.

### 4.4. Macrosystem: Integrating Filial Piety Culture With Care Requirements

At the macrosystem level, the culture of filial piety in China has a profound impact on disability management. It highlights family‐based care as a moral duty rooted in Confucian ethics, which makes carers view providing care for older adults as an indisputable duty [61]. However, this cultural expectation can also increase carer burden, restrict the use of formal services and lead to conflict between traditional values and modern care realities [62]. Finding a balance between filial obligation and external support is crucial. As a modern method of fulfilling filial duties, policies should encourage the incorporation of professional resources into care systems. Culturally sensitive interventions, such as incorporating family members into care teams and providing tailored training, can reconcile traditional values with modern demands. This establishes an ethically sound and practically viable system for caring for those with disabilities. Besides, rapid urbanisation and large‐scale internal migration [63] have weakened traditional coresidence patterns, making it increasingly difficult for adult children to provide hands‐on daily care. Many older adults now live alone or are geographically separated from their children, creating a growing need for remote, technology‐mediated disability management solutions such as tele‐rehabilitation [64], digital monitoring [65], and carer support. These shifts highlight the importance of integrating modern care technologies with culturally rooted expectations of filial responsibility.

### 4.5. Implications for Nursing Management

There are several implications for nursing management from this study. First, make functional ability a routine nursing observation. Nurse managers should shift attention from disease to function by embedding brief IC screening (mobility, self‐care and cognition) into admission assessment, discharge planning and community follow‐up and by treating any change in function as a handover item equal to a vital sign. This may help turn ‘disability literacy’ from an abstract aim into a measurable, repeatable nursing act. Second, establishing a caregiver skills training program led by specialised nurses can provide informal caregivers with structured training—including safe transfers, repositioning, basic rehabilitation exercises, medication safety, and early recognition of disease progression. Such training (including content delivered via telemedicine) can enhance caregivers’ capabilities and alleviate their burden [66]. In China, such training can be implemented through ‘Internet Plus Nursing Services’, extending specialised nursing services to the home. Third, within the context of China’s integrated healthcare systems, nursing managers should take the lead in establishing shared referral and follow‐up pathways, setting up nurse‐led transition care clinics and assigning a ‘nursing transition coordinator’ to bridge the gap in care following discharge. Incorporating older adults and family caregivers into a single, shared care plan can prevent the loss of information that currently occurs at the boundaries of the system. Fourth, in a hospital setting, CNS should not merely be consulted when needed on an ad hoc basis; rather, they should be permanent members of multidisciplinary teams and serve as case managers, coordinating assessments, goal setting and interventions around shared functional goals. Fifth, emotional support and recognition of the caregiver’s role are core elements of specialised nursing interventions [66]. Nursing managers should include family caregivers in the integrated family care team and have mental health or psychiatric nurse specialists conduct routine screenings of caregivers’ burden and psychological well‐being, while ensuring they have easy access to respite care. Finally, efforts should be made to advocate for the establishment of staffing standards and skill sets for primary care providers and to promote the integration of disability management with China’s long‐term care insurance pilot programs.

### 4.6. Limitations

Several limitations should be considered when interpreting the findings. Firstly, the study was limited to one region in eastern China, despite the use of maximum variation sampling. It may limit the transferability of the findings to other socioeconomic or regional contexts, particularly rural areas and western regions. Secondly, while efforts were made to include older adults with a range of disabilities, those with severe cognitive or communication impairments were excluded. This may have resulted in the views of the most vulnerable subgroups being overlooked. Thirdly, the predominance of female carers in our sample reflects gendered caregiving norms in China but may have underrepresented the unique challenges faced by male carers, particularly in the context of filial piety, where men may experience different role expectations, stigma or barriers to help‐seeking. Additionally, the method of recruiting carers through older adults already enrolled in the study may have further biased the sample toward dyads with more stable relationships or better communication. Consequently, the unique experiences of those in strained caregiving relationships may not be fully represented in the sample. Finally, despite the richness of the qualitative approach, it is not possible to quantify the strength or prevalence of the identified themes or to statistically meaningfully explore subgroup differences (e.g., by carer type or disability level).

## 5. Conclusion

Disability management in older adults represents a complex, dynamic interplay among individuals, informal care networks and structural health systems. By applying a multistakeholder ecological lens, this study emphasises that systemic fragmentation in disability management is not merely a consequence of institutional resource deficits but is deeply anchored in a shared disability literacy deficit and culturally reinforced care dependencies. In particular, our findings challenge the conventional reliance on familial moral obligation by revealing the paradoxical nature of filial piety: While it serves as the cultural backbone of long‐term care, it also masks care needs and traps family carers in physical and psychological strain. Crucially, this study reorients disability management from a fragmented, reactive model to an interdependent, function‐oriented continuum of care. To operationalise this paradigm shift within ageing societies, specialised nurses must be empowered as pivotal systemic coordinators who can bridge institutional chasms between hospitals and communities, translate functional preservation into everyday family caregiving and align formal healthcare resources with culturally sensitive home practices. Future research and health policy should prioritise integrated, multilevel frameworks that embed routine functional assessments and targeted caregiver capacity‐building into the core of sustainable healthy ageing strategies.

## Author Contributions

Concept and design: Yitong Mao and Zhiting Guo. Collection and assembly of data: Yitong Mao and Zhiting Guo. Data analysis and interpretation: All authors.

## Funding

This paper was developed with funding from the Scientific Research Fund of National Health Commission of the People’s Republic of China (WKJ‐ZJ‐2406).

## Disclosure

All authors gave final approval of the manuscript. All authors agree to be accountable for all aspects of the work. The funder had no input into the design, methods, analysis or interpretation of results.

## Conflicts of Interest

The authors declare no conflicts of interest.

## Supporting Information

Additional supporting information can be found online in the Supporting Information section.

## Supporting information


**Supporting Information** The supplement data can be accessed online, including Consolidated Criteria for Reporting Qualitative Research (COREQ): a 32‐item checklist (Appendix S1), the interview guide (Appendix S2) and themes and representative quotes of the participants (Appendix S3).

## Data Availability

The data that support the findings of this study are available on request from the corresponding author. The data are not publicly available due to privacy or ethical restrictions.
