# Supplementary material for: ‘It Should Be All Hands on Deck’: A Qualitative Exploration of Shared Experiences and Needs of Older Adults, Family Carers, and Health Professionals in Disability Management in China
Source: J Nurs Manag. 2026 Oct 11;2026:6577817. doi: 10.1155/jonm/6577817 (PMC13655497; doi:10.1155/jonm/6577817)
Supplement: Supplementary file 1 [file JONM-2026-6577817-s001.zip › Consolidated criteria for reporting qualitative research (COREQ) 32-item checklist.docx]

**Consolidated criteria for reporting qualitative research (COREQ): 32-item checklist**

| **No. Item** | **Guide questions/description** | **Section reported** |
| --- | --- | --- |
| **Domain 1: Research**  **team and reflexivity** |  |  |
| *Personal Characteristics* |  |  |
| 1. Interviewer/facilitator | Which author/s conducted the interview or focus  group? | Methods |
| 2. Credentials | What were the researcher’s credentials? (e.g., PhD, MD) | Methods |
| 3. Occupation | What was their occupation at the time of the study? | Methods |
| 4. Gender | Was the researcher male or female? | Methods |
| 5. Experience and training | What experience or training did the researcher have? | Methods |
| *Relationship with participants* |  |  |
| 6. Relationship established | Was a relationship established prior to study commencement? | Methods |
| 7. Participant knowledge of the interviewer | What did the participants know about the  researcher? (e.g., personal goals, reasons for doing the research) | Methods |
| 8. Interviewer characteristics | What characteristics were reported about the interviewer/facilitator? (e.g., Bias, assumptions, reasons and interests in the research topic) | Methods |
| **Domain 2: Study design** |  |  |
| *Theoretical framework* |  |  |
| 9. Methodological orientation and theory | What methodological orientation was stated to underpin the study? (e.g., grounded theory, discourse analysis, ethnography, phenomenology, content analysis) | Methods |
| *Participant selection* |  |  |
| 10. Sampling | How were participants selected? (e.g., purposive, convenience, consecutive, snowball) | Methods |
| 11. Method of approach | How were participants approached? (e.g., face-to-face, telephone, mail, email) | Methods |
| 12. Sample size | How many participants were in the study? | Results |
| 13. Non-participation | How many people refused to participate or dropped out? Reasons? | N/A |
| *Setting* |  |  |
| 14. Setting of data collection | Where was the data collected? (e.g., home, clinic, workplace) | Methods |
| 15. Presence of non-participants | Was anyone else present besides the participants and researchers? | Methods |
| 16. Description of sample | What are the important characteristics of the sample? (e.g., demographic data, date) | Results |
| *Data Collection* |  |  |
| 17. Interview guide | Were questions, prompts, guides provided by the authors? Was it pilot tested? | Methods |
| 18. Repeat interviews | Were repeat interviews carried out? If yes, how many? | No, Methods |
| 19. Audio/visual recording | Did the research use audio or visual recording to collect the data? | Methods |
| 20. Field notes | Were field notes made during and/or after the interview or focus group? | Methods |
| 21. Duration | What was the duration of the inter views or focus group? | Methods |
| 22. Data saturation | Was data saturation discussed? | Methods |
| 23. Transcripts returned | Were transcripts returned to participants for comment and/or correction? | Methods |
| **Domain 3: Analysis and findings** |  |  |
| *Data analysis* |  |  |
| 24. Number of data coders | How many data coders coded the data? | Methods |
| 25. Description of the coding tree | Did authors provide a description of the coding tree? | N/A |
| 26. Derivation of themes | Were themes identified in advance or derived from the data? | Methods |
| 27. Software | What software, if applicable, was used to manage the data? | Methods |
| 28. Participant checking | Did participants provide feedback on the findings? | No |
| *Reporting* |  |  |
| 29. Quotations presented | Were participant quotations presented to illustrate the themes/findings? Was each quotation identified? (e.g., participant number) | Results |
| 30. Data and findings consistent | Was there consistency between the data presented and the findings? | Results |
| 31. Clarity of major themes | Were major themes clearly presented in the findings? | Results |
| 32. Clarity of minor themes | Is there a description of diverse cases or discussion of minor themes? | Results |
