# Supplementary material for: ‘It Should Be All Hands on Deck’: A Qualitative Exploration of Shared Experiences and Needs of Older Adults, Family Carers, and Health Professionals in Disability Management in China
Source: J Nurs Manag. 2026 Oct 11;2026:6577817. doi: 10.1155/jonm/6577817 (PMC13655497; doi:10.1155/jonm/6577817)
Supplement: Supplementary file 1 [file JONM-2026-6577817-s001.zip › Supplementary data.docx]

**“It should be all hands on deck.”: A Qualitative Exploration of Shared Experiences and Needs of Older Adults, Family Carers, and Health Professionals in Disability Management in China**

| **Name** | **Contents** | **Page** |
| --- | --- | --- |
| Appendix S1 | Consolidated criteria for reporting qualitative research (COREQ): 32-item checklist. | 2 |
| Appendix S2 | The interview guide. | 6 |
| Appendix S3 | Themes and representative quotes of the participants. | 8 |

**Appendix S1. Consolidated criteria for reporting qualitative research (COREQ): 32-item checklist**

| **No. Item** | **Guide questions/description** | **Section reported** |
| --- | --- | --- |
| **Domain 1: Research**  **team and reflexivity** |  |  |
| *Personal Characteristics* |  |  |
| 1. Interviewer/facilitator | Which author/s conducted the interview or focus  group? | Methods |
| 2. Credentials | What were the researcher’s credentials? (e.g., PhD, MD) | Methods |
| 3. Occupation | What was their occupation at the time of the study? | Methods |
| 4. Gender | Was the researcher male or female? | Methods |
| 5. Experience and training | What experience or training did the researcher have? | Methods |
| *Relationship with participants* |  |  |
| 6. Relationship established | Was a relationship established prior to study commencement? | Methods |
| 7. Participant knowledge of the interviewer | What did the participants know about the  researcher? (e.g., personal goals, reasons for doing the research) | Methods |
| 8. Interviewer characteristics | What characteristics were reported about the interviewer/facilitator? (e.g., Bias, assumptions, reasons and interests in the research topic) | Methods |
| **Domain 2: Study design** |  |  |
| *Theoretical framework* |  |  |
| 9. Methodological orientation and theory | What methodological orientation was stated to underpin the study? (e.g., grounded theory, discourse analysis, ethnography, phenomenology, content analysis) | Methods |
| *Participant selection* |  |  |
| 10. Sampling | How were participants selected? (e.g., purposive, convenience, consecutive, snowball) | Methods |
| 11. Method of approach | How were participants approached? (e.g., face-to-face, telephone, mail, email) | Methods |
| 12. Sample size | How many participants were in the study? | Results |
| 13. Non-participation | How many people refused to participate or dropped out? Reasons? | N/A |
| *Setting* |  |  |
| 14. Setting of data collection | Where was the data collected? (e.g., home, clinic, workplace) | Methods |
| 15. Presence of non-participants | Was anyone else present besides the participants and researchers? | Methods |
| 16. Description of sample | What are the important characteristics of the sample? (e.g., demographic data, date) | Results |
| *Data Collection* |  |  |
| 17. Interview guide | Were questions, prompts, guides provided by the authors? Was it pilot tested? | Methods |
| 18. Repeat interviews | Were repeat interviews carried out? If yes, how many? | No, Methods |
| 19. Audio/visual recording | Did the research use audio or visual recording to collect the data? | Methods |
| 20. Field notes | Were field notes made during and/or after the interview or focus group? | Methods |
| 21. Duration | What was the duration of the inter views or focus group? | Methods |
| 22. Data saturation | Was data saturation discussed? | Methods |
| 23. Transcripts returned | Were transcripts returned to participants for comment and/or correction? | Methods |
| **Domain 3: Analysis and findings** |  |  |
| *Data analysis* |  |  |
| 24. Number of data coders | How many data coders coded the data? | Methods |
| 25. Description of the coding tree | Did authors provide a description of the coding tree? | N/A |
| 26. Derivation of themes | Were themes identified in advance or derived from the data? | Methods |
| 27. Software | What software, if applicable, was used to manage the data? | Methods |
| 28. Participant checking | Did participants provide feedback on the findings? | No |
| *Reporting* |  |  |
| 29. Quotations presented | Were participant quotations presented to illustrate the themes/findings? Was each quotation identified? (e.g., participant number) | Results |
| 30. Data and findings consistent | Was there consistency between the data presented and the findings? | Results |
| 31. Clarity of major themes | Were major themes clearly presented in the findings? | Results |
| 32. Clarity of minor themes | Is there a description of diverse cases or discussion of minor themes? | Results |

**Appendix S2. The interview guide**

**Research questions**

***For older adults***

1. What is your opinion about disability? How do you feel about disability?

2. What bothers you the most in your daily life?

3. What measures have you taken to prevent or intervene disability? And their effect?

4. What are your expectations and suggestions for disability prevention and intervention measures at home?

5. Is there anything you would like to add?

***For health professionals***

1. What is your opinion about disability?

2. What measures have you taken for the older adults to prevent or intervene disability in your department/community? And their effect?

3.What are the main tasks of hospital/community health center in preventing and managing disability in older adults?

4. What are the challenges and problems of existing measures?

5. Is there anything you would like to add?

***For family carers***

1. What is your opinion about disability?

2. What difficulties have you encountered in caring for older adults?

3. What measures have you taken for the older adults to prevent or intervene disability? And their effect?

4. What are your suggestions for disability prevention and intervention at home?

5. Is there anything you would like to add?

**Appendix S3. Themes and representative quotes of the participants.**

| **Themes** | **Representative quotes** |
| --- | --- |
| (1) Knowledge and attitude gaps regarding disability | *“Eat less, exercise more, because there is starch in rice; eat less, exercise more… If you want to prevent (disability), you can run, exercise, communicate with others, and talk to them.”* *(C2, older adult, mild disability)*.  *“If this is done well, it will have a very positive impact on the health of the older adults and their quality of life, such as life expectancy, and their ability to take care of themselves.” (B5, a community chief nurse).*  *“In fact, a general hospital like us may still be primarily a diagnostic and therapeutic aspect of it (disability).” (A6, a* *hospital* *physician)*.  *“Because like what we can understand is basic wound care and exercise, um, and then (further), like the equipment type, like to build up the muscles and to what (extent), maybe we don't know very much about that, so there's no way to (instruct the older adults and their families).” (B4, a community nurse).* |
| (2) Excessive care burden | *“My eyes look so haggard now. These marks (pointing to the area around her eyes) are from wearing glasses. I didn’t have any wrinkles before, but they’ve all appeared over the past four years. Look how pathetic I am; I can't go anywhere, only stay at home to buy vegetables and eggs, very busy…Sigh, there’s just nothing we can do.” (D9, spouse of an adult with moderate disability)*  “*It's not easy for her to bring up a child. She has to take him to school early in the morning and pick him up in the evening, which is far away from us. She cooks dinner and tutors the child, so she is very busy…She does all the housework.” (C4, older adult, moderate disability).* |
| (3) Care coordination between home and hospital | *“If there is ongoing care, there is a service that extends to him. This means that even after the patient is discharged, we will continue to monitor their condition. It does not mean that we will stop checking in once treatment is complete—nor should we. Many of our patients are repeatedly hospitalized.” (A4, a hospital chief nurse).*  *“(After discharge) a continuous follow-up is needed. For example, discharge planning services are designed to determine where each patient will go after discharge. As I have stated, if disabled older adults need ongoing rehabilitation and care after returning home, the community must bear responsibility for this. What we've done is not yet ideal.” (A8, a hospital physician).* |
| (4) Interdisciplinary professional team-building | *“The geriatrics department needs to provide holistic and comprehensive care for the older adults, especially the disabled, so it should be a combination of various disciplines and a very strong team of senior doctors.” (A4).*  *“They will then issue the appropriate medical recommendations, and we will provide health education and guidance. After that, we will review (the results) and provide feedback to the doctor.” (A2, a hospital nurse).*  *“At present, we have only two specialist geriatric nurses. We plan to create additional roles, such as palliative care nurses. As there are many patients in the geriatrics department who require palliative care, and given the need for nutritional care, we also plan to provide training for these specialist nurses in both areas.” (A9, a hospital nurse).*  *“If the team members are familiar with each other, everyone will have a better anticipation of the disease treatment.” (A1,* *a community nurse)* |
| (5) Community resource allocation | *“Due to limited staff resources, the rate of early identification of disabilities is not particularly high. Even when we carry out home visits, we only visit once a month.” (B1, a community physician)*. |
| (6) Involvement of social resources | *“As for the management of disability, the government currently has some policies in place, but they need to be further promoted. I think this is something we can think about in the future.” (B5)* |
| (7) The dual influence of filial piety culture | *“Because I’m her daughter, if I don’t look after her, who will? There’s no one else to look after her.” (D16, spouse of an adult with moderate disability)* |
|  | *“My siblings and I take turns looking after our father. If we were to send him to a care home, people might think we are being unfilial. After all, as their children, we aren’t looking after him ourselves.” (D15, child of an adult with severe disability)* |
